# Heterosexual behaviours among men who sell sex to men in coastal Kenya

**DOI:** 10.1097/QAD.0000000000000889

**Published:** 2015-12

**Authors:** Adrian D. Smith, Allan D. Muhaari, Carole Agwanda, Dickens Kowuor, Elise van der Elst, Alun Davies, Susan M. Graham, Harold W. Jaffe, Eduard J. Sanders

**Affiliations:** a Nuffield Department of Population Health, University of Oxford, Oxford, UK; b Kenya Medical Research Institute-Wellcome Trust Research Programme, Kilifi, Kenya; c Department of Medicine, Global Health and Epidemiology, University of Washington, Seattle, Washington, USA; d Centre for Topical Medicine and Global Health, Nuffield Department of Medicine, University of Oxford, Oxford, UK; e Department of Global Health, Academic Medical Centre, University of Amsterdam, Amsterdam, the Netherlands

**Keywords:** diary study, Kenya, male sex work, men who have sex with men, MSM sex workers, sexual behaviour

## Abstract

**Objective:**

African men who have sex with men often sell sex to men, and MSM who sell sex (MSM-SW) often also have female partners. We compared sexual risk behaviour of MSM-SW who were sexually active with female partners (bisexual MSW) to MSM-SW with only male partners (exclusive MSW).

**Design:**

Descriptive behavioural study

**Methods:**

A novel, validated daily event and partner diary self-completed by 82 MSM who sold sex over a follow-up period of 42 days with weekly review. Cumulative individual counts of sex and condomless sex were compiled by partner characteristics. The incidence of specific partnerships and sex acts were compared within and between bisexual and exclusive MSW.

**Results:**

Most (59%) MSM-SW reported female partners during follow-up. The majority of both male and female partners were cash-paying clients originating locally. Bisexual MSW reported a similar rate of condomless sex with male and female partners, but significantly fewer male partners than exclusive MSW. Bisexual MSW had lower HIV prevalence, were more likely to only report insertive anal sex roles, and reported lower frequencies of condomless receptive anal sex than exclusive MSW.

**Conclusion:**

Bisexually active male sex workers in coastal Kenya create HIV and other sexually transmitted infection transmission pathways to partners and clients in both MSM and heterosexual networks, but differed from exclusive MSW in having lower HIV acquisition and transmission risks. Epidemiological projection methods are liable to overestimate bridging potential of MSM-SW and MSM populations without account for systematic differences in risk within these populations.

## Background

Reviews of HIV prevalence and incidence studies demonstrate the high HIV burden and unmet need for HIV care among men who have sex with men (MSM) populations in all sub-Saharan settings where studies have been undertaken [1,2]. Transactional activities are commonly documented by men recruited to MSM surveys in Africa, and in these surveys MSM reporting recent sex work often have a higher HIV prevalence than MSM who do not [3–9]. MSM sex workers (MSM-SW) – MSM who sell sex to men in exchange for money – have received comparatively little specific attention in sub-Saharan Africa [10]. Elsewhere, high HIV acquisition risks among MSM-SW reflect high male partner frequencies and the high biological HIV transmission risk associated with receptive anal sex practices [11–13].

A number of studies of male sex work and HIV risk have been reported from urban settings in Kenya [14–16]. Kenya has a declining generalized HIV epidemic with well characterized concentrated epidemics among key populations, including MSM [17,18]. Studies of MSM-SW in Nairobi estimate HIV prevalence at 26.3% (respondent driven sample) [15] and 40.0% (convenience sample) [14], and a high HIV incidence (10.9 per 100 person-years) [14]. Retrospective behavioural surveys suggest that many MSM-SW report female partners (recall period: 3 month 37.6% [14], 12 month 49.1% [15], lifetime 73.6% [19]), leading researchers and policymakers to speculate about a potential transmission bridging role of MSM-SW between MSM and heterosexual networks [14,15,20].

Mode of transmission models are used across Africa to project the population fraction of new HIV infections attributed to different risk groups using estimates of population sizes, risk behaviour and HIV prevalence, and thereby to assign HIV prevention and control resources effectively [23]. In coastal Kenya, HIV prevalence among MSM is greater than among the general adult population (MSM: 18.2% [18], women: 6.2% [22], men: 2.2% [22]). Mode of transmission studies project that 11% of all new HIV infections in coastal Kenya arise among MSM (outside prison environments) and, assuming that half of MSM also have female sexual partners, a further 2% of all new HIV infections occur among female partners of MSM [20]. However, this projection method also implicitly assumes that HIV prevalence and behavioural risk among MSM who have female sexual partners is otherwise no different from MSM who do not.

In this study we describe prospectively ascertained sexual partners and behaviours of MSM-SW drawn from a well established key population cohort study in Coastal Kenya, and compare sexual behaviour of MSM-SW who report female partnerships with those who do not.

## Methods

### Study site and population

The prospective diary study was nested in two ongoing cohort studies of adults at high risk of HIV infection, or with known HIV infection, in Mtwapa, Kenya [21,24]. In brief, these studies recruited adults considered to be at high risk of HIV infection, including MSM, into follow-up. MSM were identified by a team of 10–15 peer mobilizers who approached individuals via personal networks and social venues. Follow-up comprised quarterly risk assessment (socio-demographic and behavioural questionnaire), clinical evaluation (symptom history, physical examination) and laboratory assessment [rapid HIV testing, sexually transmitted infection (STI) diagnostics].

All MSM cohort participants who reported sex with another man in the previous 3 months at their most recent risk assessment were invited to participate in the diary study. Diary study volunteers provided written informed consent to participate.

### Ethical approval

Ethical approval for this study was granted by the Kenya Medical Research Institute Ethics Review Board and the Oxford Tropical Research Ethics Committee, University of Oxford.

### Diary instrument

**Fig. 1:**
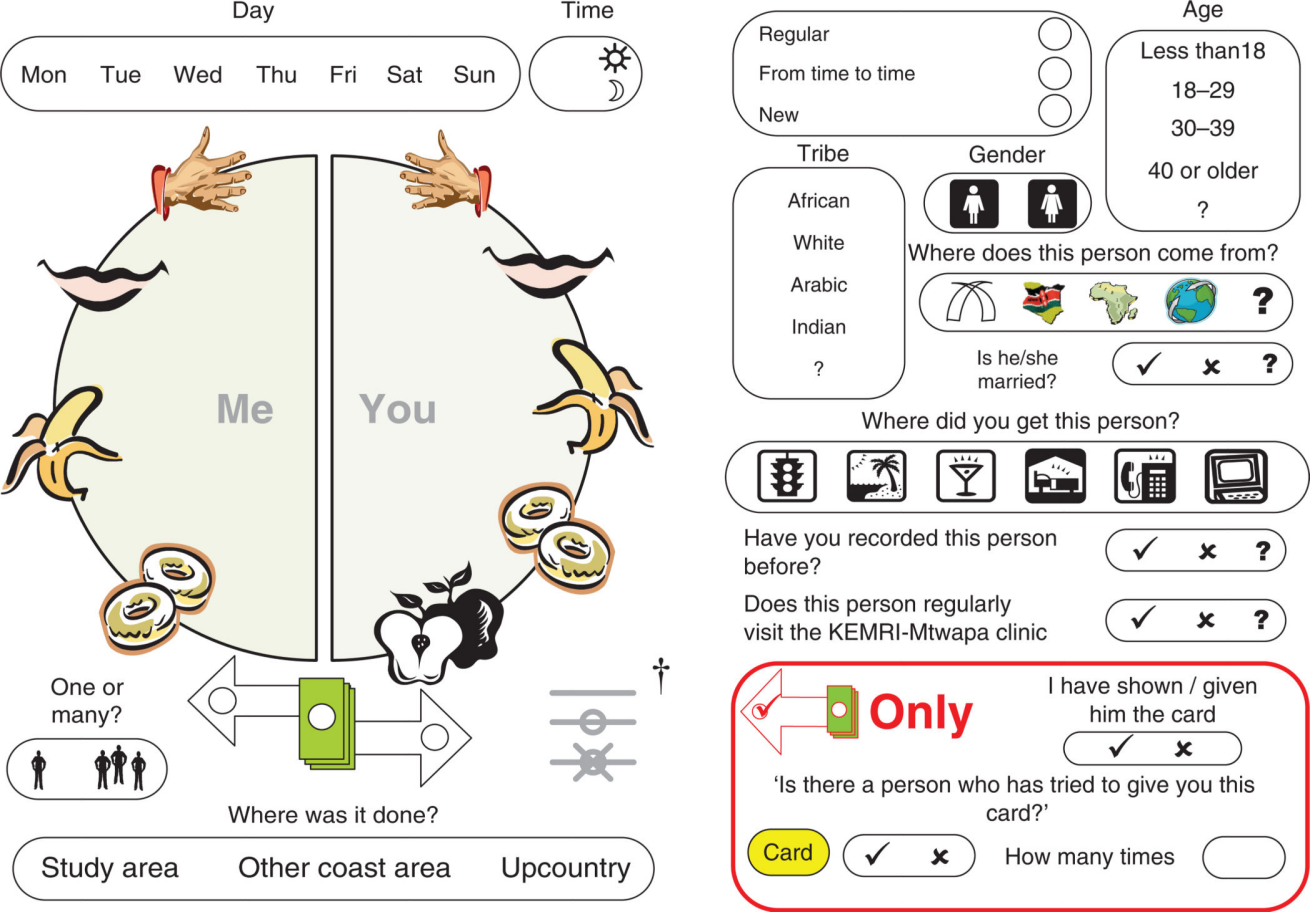
Event diary instrument (back-translated from Kiswahili) Sexual acts recorded by joining the symbolic body part of the participant (*left-hand side of disc*, ‘*me*’: symbols for hand, mouth, penis (banana), anus (donut) to symbolic body part of partner (*right-hand side of disc*, ‘*you*’ including vagina (apple). Multiple lines indicate repeated rounds of a sexual act. ^†^Lines appended for condom use and breakage for each round indicated. Partner information (right) included sex, age, ethnicity (tribe), marital status and location of origin [Mombasa (tusks), Kenya, Africa, or international].

Event diaries were developed in a pilot programme over 6 months with a group of MSM volunteers. The self-completed event diary was set in pocket book form for ease of carriage and allowed space for 27 consecutive event entries. Sexual acts with each partner were documented using a pictographic disc (Fig. 1, back translated from Kiswahili). Diary keepers recorded specific sexual acts by drawing a line between body parts of themselves and their partners. Body parts were represented as icons chosen to be meaningful to trained diary users, but not to be interpretable or incriminating if found by someone else. Condom use was indicated by annotation of connecting lines. Characteristics of the sexual partner (e.g. sex, age group, ethnicity, residential origin, and marital status) were recorded by the diary keeper, with an option to indicate if a response was unknown. Recorded characteristics of the dyad included visual coding of cash exchanged for sex and the type of partnership. Regular partners were defined as steady boyfriends, girlfriends and spouses. Casual partners included new partners (defined as partners encountered for the first time) and casual recurrent partners [defined as recurrent partners not considered to be regular partners and back-translated from the Kiswahili *mara kwa mara* (‘from time to time’, Fig. 1)].

Participants completed a 4-day training on use of the diary, followed by a 3-week lead-in period during which they were required to demonstrate competence and accuracy in recording. Participants who met this requirement completed a questionnaire at study entry, including data on socio-demographic factors and lifetime sexual behaviour, then were issued with diaries, condoms and lubricants. Thereafter participants completed diaries for 42 consecutive days each during October–December 2007, depositing completed diaries each week. Event diaries demonstrated high agreement with contemporaneous cohort measures, strong user acceptability in exit evaluation, and high predictive validity against subsequent HIV incidence through January 2011 [25].

### Data management and analysis

Diary entries were error-checked with participants at weekly collection, then double-entered using a pictographic abstraction program (Microsoft FoxPro). Following abstraction, event diaries were archived securely.

Analysis was limited to 82 MSM who reported any sex with a man who paid for sex with cash in the diary study (MSM-SW). One MSM participant did not report selling sex, and was excluded from analysis. We defined behaviourally bisexual MSM-SW (biMSW) as MSM-SW who reported sex with male and female partners and behaviourally exclusive MSM-SW (exMSW) as MSM-SW who reported only male partners during the diary study. Baseline sociodemographic characteristics of biMSW and exMSW were compared using Fisher’s exact test or Wilcoxian rank-sum [26].

Discrete event records were coded to dichotomous variables for each sex act – insertive anal intercourse (IAI), receptive anal intercourse (RAI) and vaginal intercourse, then by condom use for each sex act. Penetrative intercourse was defined as an event in which one or more anal or vaginal acts occurred. Condomless sex was defined as an intercourse event including one or more condom-unprotected acts. The condomless event probability was the fraction of occurrences of a sex act that were condomless. Chi-squared tests were used for direct comparison of proportions (χ^2^).

Event data were compiled into summary counts for each participant, specifically the count of sex acts and condomless sex acts per individual. The incidence of sex acts was described as the average count of sexual acts per 4 weeks of follow-up. Count distributions were strongly positively skewed with best fit to the negative binomial distribution assessed against Poisson, zero-inflated Poisson and zero-inflated negative binomial [27].

To compare sexual behaviour frequency of different participants we used negative binomial regression to estimate the crude incidence rate ratio (IRR) of partner, sex act and condomless sex act frequency by participant covariates. Generalized estimating equations (GEE) with a logit link function and exchangeable correlation matrix were used to estimate the odds ratio (OR) of condomless intercourse by participant characteristics, adjusting for correlation of repeated events per participant. Multivariate models were used to estimate independent associations (aIRR and aOR) between participant category and sexual behaviour, adjusted for other participant covariates (HIV status and age). To compare within-individual intercourse and condomless intercourse frequencies, we used GEE with a negative binomial link for panels of paired count measures to estimate the crude within-individual IRR.

## Results

Eighty-two diary participants reported at least one male sexual partner who paid cash for sex over an average follow-up of 41.2 days. At study entry, 65 (79.3%) MSM-SW reported previous sex with female partners before the study. Forty-eight (58.5%) MSM-SW reported sex with female partners in event diaries (biMSW) and 34 (41.5%) MSM-SW reported sex with only men (exMSW).

### Participant characteristics

Table 1 reports selected characteristics of MSM diary study participants who reported selling sex to men during prospective follow-up. Thirty-nine percentage of MSM-SW were under the age of 25. BiMSW were older and more likely to report previous marriage than exMSW, yet were no more likely to presently be married. MSM-SW reported low levels of formal employment and income (2000 KSH/month roughly equivalent to USD$1/day), yet no differences in participant education, employment, income or religious affiliation were noted between biMSW and exMSW. BiMSW were markedly less likely to be HIV seropositive than exMSW (8.3 and 50.0% respectively, *P* < 0.001).

### Differences in male–male sexual behaviour between exclusive and bisexual MSW

MSW diary participants reported 1386 sexual events with men. About 94.4% events included anal intercourse (Table 2) whilst 5.5% involved only oral sex or masturbation. On average, biMSW reported 30% fewer anal intercourse events with male partners than did exMSW (representing four fewer instances of anal sex per 4 weeks per person), a lower rate of condomless anal sex (representing one less instance of condomless sex per 4 weeks per person), but a similar event probability of condomless anal intercourse.

BiMSW reported lower rates of anal intercourse with men than did exMSW in all transaction and relationship categories; however, patterns of male partnership were very similar. BiMSW reported significantly fewer male partners who paid cash for sex than did exMSW (Table 2), but for both groups, paying partners were the vast majority of male sexual contacts (biMSW: 89.1%; exMSW 88.3%, χ^2^*p* = 0.877) and condomless male sexual contact (biMSW: 85.8%; exMSW: 82.9%, χ^2^*p* = 0.877). Anal sex acts with men who paid cash for sex were less often condomless than sex with nontransactional male partners [biMSW OR: 0.52, 95% confidence interval (CI) 0.23–1.19, *P* = 0.124; exMSW OR 0.52, 95% CI 0.22–1.22, *p* = 0.133, neither statistically significant]. Purchase of sex from other men was infrequently reported by biMSW or exMSW.

Relationship patterns differed somewhat for biMSW and exMSW. Bisexual MSW reported lower rates of anal intercourse and condomless anal intercourse with repeat male partners (regular or casual recurrent), but a similar rate of anal intercourse and higher rate of condomless anal intercourse with new partners (Table 2). Overall, 55.7% (102/183) of condomless sex with men reported by biMSW was with new partners, whilst most condomless sex reported by exMSW was with recurrent partners (65.2%, 118/181). There were no significant differences in condomless event probability between biMSW and exMSW by relationship category; however, sex with regular male partners was more likely to be unprotected than sex with casual partners for both biMSW (OR 3.43, 95% CI 1.32–8.28, *p* = 0.006) and exMSW (OR 2.93, 95% CI 1.49–5.77, *p* = 0.002).

Differences in anal intercourse role with male partners were marked. BiMSW reported lower rates of RAI than IAI (within-individual IRR 0.30, 95% CI 0.19–0.46, *P* < 0.001), whereas exMSW reported the reverse (within-individual IRR 2.23, 95% CI 1.46–3.43, *P* < 0.001). Compared to exMSW, biMSW were much more likely to ‘only’ report IAI with men throughout follow-up (biMSW: 50% vs. exMSW: 11.8%, Fisher’s exact *P* < 0.001).

BiMSW reported significantly lower rates of RAI and condomless RAI than exMSW (Table 2). These associations were independent of the pronounced negative association between increasing participant age and RAI frequency, and strong associations between participant HIV status and RAI and condomless RAI frequency (Table 3). BiMSW did report somewhat higher frequencies of IAI and condomless IAI than exMSW (Table 2); however, there was no independent association after adjustment for the prominent negative associations between IAI frequency, condomless IAI frequency and participant HIV status (Table 3).

Similarly, MSM-SW who recalled sex with females prior to the diary study similarly reported lower prospective frequencies of RAI (aIRR 0.35, 95% CI 0.16–0.80, *p* = 0.001) and condomless RAI (aIRR 0.19, 95% CI 0.06–0.58, *P* = 0.003), and higher frequencies of IAI (aIRR 2.65, 95% CI 1.60–4.37, *P* < 0.001) and condomless IAI (aIRR 2.08, 95% CI 0.68–6.40, *p* = 0.202) than MSM-SW who had never previously had sex with a female partner.

### Sexual partners and behaviour of bisexual MSW

Bisexually active MSM-SW reported details of 214 female and 510 male partners. Male partners were often in an older age group than participants (male 49.5, female 23.5%, χ^2^*p* < 0.001). Most partners were resident in Kenya (male 92.3, female 92.1%) and of African ethnicity (male 63.9, female 79.3%). Male partners were more likely to be described as Arabic/Indian than female partners, whereas a minority of both were described as Whites (male 4.3, female 3.3%). Male partners were more often believed to be married (male: 42.0; female 31.0%), χ^2^ *P* = 0.017) though this detail was often unknown (30.4%).

Table 4 summarizes rates of penetrative intercourse and condomless sex among biMSW with their male and female partners. In addition to intercourse with male partners discussed above, all biMSW reported vaginal intercourse and 79.2% also reported anal sex with a female partner. In 340 intercourse reports with female partners, 49.1% were vaginal intercourse only, 10.4% were anal intercourse only and 40.0% were both. Condom use did not differ for anal and vaginal intercourse with women (Table 4).

BiMSW reported approximately twice as much penetrative intercourse with men as with women (9.1 vs. 4.8 acts per 4 weeks, IRR 1.83, 95% CI 1.39–2.40, *P* < 0.001, Table 4). This difference consisted of significantly higher intercourse rates with casual and cash-paying male partners. By contrast, more nontransactional intercourse was reported with female rather than male partners, and the vast majority of sex paid for by the participant was with women. A similar proportion of biMSW reported female (47.9%) and male (54.2%) regular sexual partners (31.2% reporting both), both accounting for similar intercourse frequencies. With both male and female partners, penetrative intercourse was most likely to be condomless with regular partners or nontransactional partners (Table 4).

Differences in rates of condomless intercourse with male and female partners were more modest than differences in intercourse frequency due to the consistently lower probability of condom use with female partners, compared to male partners (Table 4). As a result, among biMSW, the overall rate of condomless intercourse with female partners was similar to that with male partners (2.1 vs. 2.6 condomless acts per 4 weeks, Table 3) and 44.5% (147/300) of all biMSW condomless acts were with female partners.

## Discussion

Over a brief duration of detailed prospective observation the majority of MSM who sold sex to men also reported sexual behaviours with women, often including anal intercourse. Sexual activity with both men and women was overwhelmingly characterized as transactional. The existence of female clients who pay for sex from male sex workers on the Kenya coast has previously been indicated by cross-sectional studies of male sex workers on the Kenya coast [16,19], and one study suggests female sex workers commonly report paying for sex with men [28]. Analysis of our findings demonstrates that demand from women represents a significant fraction of male sex work activity, originates mostly from local women and that sex is frequently condomless. These findings both challenge the prevailing characterization of such demand coming from international tourists [29], and highlights the need to investigate a very poorly understood aspect of sexual culture of potential significance to HIV and STI control in Kenya.

Bisexually active MSM-SW reported higher frequencies of sex with male than female partners, but intercourse events with men were much more likely to be condomprotected than those with women. Consequently, overall rates of condomless sex with male and female partners were similar. This suggests ample potential routes of sexual transmission risk of HIV (or other STIs) between biMSW and partners of either sex. From a sexual network perspective, that most MSM-SW report active sexual behavioural links within both MSM and heterosexual sexual networks infers the potential for this group to act as a bridging population between those networks.

These data, however, also suggest that male sex workers who interact with both MSM and heterosexual networks have considerably lower behavioural HIV acquisition risk than those who interact exclusively within MSM networks. HIV prevalence among bisexually active MSM sex workers was markedly lower than among exclusive MSM sex workers, although still higher than estimated prevalence in the local adult male population [22]. These findings concur with a lower HIV incidence reported among bisexually active MSM in the cohort study from which diary study participants were recruited [21]. Similarly, pooled findings of wider sub-Saharan African MSM data suggest that both bisexually identifying and behaviourally bisexual MSM have approximately half the HIV prevalence of other MSM [30].

The above study suggests that heterogeneity in HIV prevalence between biMSW and exMSW reflects heterogeneity in same-sex risk behaviour. BiMSW had fewer male partners, were more likely to take only the insertive role with male partners, and had a significantly lower incidence of RAI and condomless RAI than did exMSW – all indicative of lower HIV acquisition risk. Further, that biMSW were less likely to report anal sex role versatility between partners, and had fewer recurrent male partners, may also suggest that biMSW may be less influential than exMSW upon dynamics of HIV transmission within MSM networks [31]. This study could not directly assess whether either higher RAI frequency or lower heterosexual activity among HIV-positive MSM-SW was in part reactive to knowledge of HIV status. However, whereas seroadaptation may well explain the lower condomless IAI rate and event probability among HIV-positive MSM-SW [32], significant negative associations between RAI and heterosexual activity were consistent across analyses restricted to HIV negative MSM-SW (data not shown), and for both prospective and lifetime measures of sex with female partners suggesting that reverse causation is an insufficient explanation for our findings.

Systematic differences in MSM HIV prevalence and acquisition risks by level of heterosexual activity may be a plausible explanation to resolve the lack of objective phylogenetic evidence of actual HIV transmission bridging across MSM and heterosexual networks to date in Kenya [33,34] with existing projections of significant bridging from models that fail to account for such heterogeneity [20]. However, to what extent patterns of risk behaviour among MSM-SW are generalizable to wider MSM populations in Kenya is uncertain. Local and regional data suggest similar disparities in risk behaviour may well exist in more broadly defined MSM populations. In coastal Kenya, qualitative studies describe Kiswahili MSM identities along very similar behavioural distinctions: *basha* describing a masculine MSM who takes the insertive sex role, has female partners and passes as heterosexual in wider society, and *shoga* that evokes notions of femininity, the receptive sex role and which is often appropriated as a term of abuse [35,36]. Few quantitative MSM behavioural studies elsewhere in sub-Saharan Africa and the diaspora report comparable data, but those that do suggest the same differentiation of anal sex role by heterosexual activity and sexual identity [11,37,38].

The study had a number of limitations. We measured only the direct sexual behaviours and contacts of MSM-SW. That many male and female partners of participants were thought to be married highlights the need for extended network data to comprehensively quantify bridging pathways. It is questionable whether such studies are feasible – our efforts to engage partners and clients of MSM-SW for research confirm that, as elsewhere [39], such groups are exceptionally elusive.

We elected to categorize MSM-SW by behaviour rather than sexual identity, having found low acceptance amongst participants of international terminology categorizing sexuality in preparatory focus groups. Behavioural categorization has the advantage of being objective and unambiguous in directly describing sexual risk, but behaviour may not correspond to personally held notions of sexuality, particularly in the context of sex work [40]. Ongoing local qualitative work concurs with observations elsewhere that bisexually active and identifying MSM are less likely to disclose same sex behaviour or access health services [1,41,42], highlighting the need to understand the relationship between behavioural risk and sexual identity in aligning sexual health responses to potentially very diverse notions of individual need and accessibility.

Although the accuracy of prospective event-level measurement in assessing HIV acquisition and transmission risk is well established [43,44], the intensity of follow-up and staff time required was considerable, limiting achievable sample size and the duration of follow-up. Further, findings may not be generalizable to MSM sex workers in other contexts. The study recruited from a research-engaged population of MSM, aware of their HIV status and with free access to relevant information, counselling and prevention materials. Entitlement to services that are otherwise expensive to access is likely to have preferentially selected for recruitment of MSM-SW on low incomes through street-based sex work, which may be unrepresentative of the full spectrum of MSM-SW working locally. ‘High-class’ MSM-SW and male escorts on high incomes are known to exist on the Kenya coast, are thought to prefer to seek care from discreet MSM-friendly private services rather than though research clinics, and may exert greater control over sexual risk in transactional interactions. Lastly, inferences are necessarily limited to men who sell sex to men – in light of findings, male sex workers with exclusively female partners and clients may well exist locally, but were not identified or included in this study.

## Conclusion

Bisexually active male sex workers in coastal Kenya create potential transmission pathways for HIV and other sexually transmitted infections to partners and clients in both MSM and heterosexual networks. However, these men are not simply MSM sex workers who happen to have female partners; rather, their behaviour differs from that of exclusive MSM sex workers in important ways that result in a lower risk of HIV infection and transmission. Modelling projections will tend to overestimate the bridging potential of MSM sex workers and other MSM populations unless they account for such risk differences.

## Figures and Tables

**Table 1:** Characteristics of study participants who reported selling sex to men during diary follow-up (prospective diary study, Coastal Kenya, 2007).

		BiMSW, *N* = 48 *N* (%) or median IQR	ExMSW, *N* = 34 *N* (%) or median IQR	*P* value (Fisher’s exact test unless indicated)
Age	Years	27 (23–32)	25.5 (22–30)	0.320^b^
Marital status	Single	34 (70.8)	30 (88.2)	0.084
	Married	5 (10.4)	3 (8.8)	
	Divorced/Widowed	9 (18.8)	1 (2.9)	
Religion	Catholic	16 (33.3)	10 (29.4)	0.841
	Protestant	14 (29.2)	8 (23.5)	
	Muslim	16 (33.3)	14 (41.2)	
	None	2 (4.2)	2 (5.9)	
Highest educational enrolment	None			0.409
	Primary	23 (47.9)	21 (61.8)	
	Secondary	19 (39.6)	11 (32.4)	
	Higher	6 (12.5)	2 (5.9)	
Employment	Formal employment	6 (12.5)	5 (14.7)	0.908
	Self-employment	19 (39.6)	14 (41.2)	
	Unemployed	23 (47.9)	15 (44.1)	
Earnings from any source (last month)	<2000 KSH	10 (20.8)	11 (32.4)	0.417
	2–5000 KSH	18 (37.5)	13 (38.2)	
	5000+ KSH	20 (41.7)	10 (29.4)	
Clinical				
Circumcised^a^	Yes	38 (84.4)	29 (87.9)	0.751
	No	7 (15.6)	4 (12.1)	
HIV status	Positive	4 (8.3)	17 (50.0)	<0.001
	Negative	44 (91.7)	17 (50.0)	

biMSW, MSM-SW who reported male and female partners during follow-up; exMSW, MSM-SW who reported only male partners during follow-up; IQR, interquartile range, KSH: Kenyan shilling.

a Data missing for four men.

b Wilcoxon rank-sum.

**Table 2:** Sexual behaviour with male partners (all MSM sex workers, prospective diary study, Coastal Kenya, 2007).

	Anal intercourse						Condomless anal intercourse							
	biMSW		exMSW		Sex frequency (biMSW vs. exMSW)		biMSW		exMSW		Condomless sex frequency (biMSW vs. exMSW)		Condomless event probability (biMSW vs. exMSW)	
	*N*	Rate	*N*	Rate	IRR† (95% CI)	*P*	*N* (%)	Rate	*N* (%)	Rate	IRR† (95% CI)	*P*	OR‡ (95% CI)	*P*
Total	642	9.1	667	13.2	0.70 (0.54–0.91)	**	183 (28.5)	2.6	181 (27.1)	3.6	0.72 (0.37–1.41)		1.06 (0.54–2.05)	
Transaction														
Clients	572	8.1	589	11.6	0.70 (0.53–0.93)	*	157 (27.4)	2.2	150 (25.4)	3.0	0.74 (0.37–1.48)		1.07 (0.54–2.15)	
Sex workers	15	0.2	18	0.4	0.60 (0.15–2.29)		2 (13.3)	<0.1	14 (77.8)	0.3	0.10 (0.01–0.92)	*	0.05 (0.06–0.44)	**
Nontransactional	55	0.8	60	1.2	0.67 (0.31–1.47)		24 (43.6)	0.3	17 (27.4)	0.3	1.01 (0.29–3.44)		1.76 (0.58–5.38)	
Relationship type														
Regular	76	1.1	86	1.7	0.63 (0.33–1.20)		27 (35.5)	0.4	35 (40.6)	0.7	0.55 (0.21–1.43)		0.96 (0.36–2.55)	
Casual – recurrent	199	2.8	235	4.7	0.61 (0.43–0.87)	**	54 (27.1)	0.8	83 (35.3)	1.6	0.47 (0.19–1.12)		0.91 (0.37–2.21)	
Casual – new	367	5.2	346	6.9	0.77 (0.55–1.08)		102 (27.8)	1.5	63 (18.2)	1.3	1.15 (0.55–2.42)		1.39 (0.69–2.80)	
Anal sex role														
Insertive	467	6.6	186	3.7	1.81 (1.22–2.70)	**	146 (31.3)	2.1	68 (36.6)	1.4	1.51 (0.61–3.76)		0.99 (0.41–2.38)	
Receptive	243	3.5	542	10.7	0.33 (0.17–0.64)	***	42 (17.3)	0.6	120 (22.1)	2.4	0.25 (0.09–0.68)	**	0.89 (0.37–2.14)	

biMSW, MSM-SW who reported male and female partners during follow-up; exMSW, MSM-SW who reported only male partners during follow-up; *N*, number of intercourse events; *p* value, Rate, average count per 4 weeks person-observation.

* *P* < 0.05.

** *P* < 0.01.

*** *P* < 0.001.

† Crude incidence rate ratio: negative binomial regression.

‡ Crude odds ratio using generalizing estimating equation grouped by individual participant with a logit link function and exchangeable correlation matrix.

**Table 3:** Adjusted associations with frequency of anal intercourse with male partners (all MSM sex workers, prospective diary study, Coastal Kenya, 2007).

		Anal intercourse			Insertive anal intercourse			Receptive anal intercourse		
		Sex frequency aIRR† (95% CI)	Condomless sex frequency aIRR† (95% CI)	Condomless event probability aOR‡ (95% CI)	Sex frequency aIRR† (95% CI)	Condomless sex frequency aIRR† (95% CI)	Condomless event probability aOR‡ (95% CI)	Sex frequency aIRR† (95% CI)	Condomless sex frequency aIRR† (95% CI)	Condomless event probability aOR‡ (95% CI)
Sexuality	biMSW	0.87 (0.67–1.12)	0.72 (0.36–1.45)	0.71 (0.19–2.66)	1.34 (0.87–2.05)	0.83 (0.33–2.13)	0.45 (0.08–2.47)	0.48* (0.27–0.89)	0.35* (0.13–0.98)	0.71 (0.16–3.11)
	exMSW	ref	ref	ref	ref	ref	ref	ref	ref	ref
Age	Per year	0.96*** (0.94–0.98)	0.94* (0.89–0.99)	0.95 (0.87–1.05)	0.99 (0.96–1.02)	0.96 (0.89–1.03)	0.88 (0.78–1.01)	0.94** (0.90–0.98)	0.96 (0.89–1.03)	1.02 (0.92–1.13)
HIV	Positive	1.54** (0.94–0.98)	0.85 (0.38–1.89)	0.28 (0.06–1.23)	0.41** (0.25–0.68)	0.09*** (0.02–0.32)	0.07* (0.01–0.69)	3.30** (1.65–6.61)	2.97* (1.04–8.46)	0.64 (0.15–2.76)
	Negative	ref	ref	ref	ref	ref	ref	ref	ref	ref

† Adjusted incidence rate ratio: multivariate negative binomial regression including tabled covariates.

‡ Adjusted odds ratio using generalizing estimating equation grouped by individual participant with a logit link function and exchangeable correlation matrix, and including tabled covariates.

* *P* < 0.05.

** *P* < 0.01.

*** *P* < 0.001.

**Table 4:** Sexual behaviours of bisexually active MSM sex workers (prospective diary study, Coastal Kenya, 2007).

	Penetrative intercourse						Condomless penetrative intercourse				Condomless sex frequency (male vs. female)		Condomless event probability (male vs. female)	
	Male partners		Female partners		Sex frequency (male vs. female)		Male partners		Female partners					
							
	*N*	Rate	*N*	Rate	IRR† (95% CI)	*P*	*N* (%)	Rate	*N* (%)	Rate	IRR† (95% CI)	*P*	OR‡ (95% CI)	*P*
Total	642	9.1	340	4.8	1.83 (1.39–2.40)	***	183 (28.5)	2.6	147 (43.2)	2.1	0.93 (0.61–1.42)		0.48 (0.37–0.61)	***
Transaction														
Partner paid	572	8.1	216	3.1	2.57 (1.87–3.53)	***	157 (27.4)	2.2	91 (42.1)	1.3	1.48 (0.89–2.44)		0.49 (0.36–0.65)	***
Participant paid	15	0.2	50	0.7	0.29 (0.14–0.60)	**	2 (13.3)	<0.1	22 (44.0)	0.3	0.09 (0.02–0.39)	**	0.16 (0.05–0.66)	*
Nontransactional	55	0.8	74	1.1	0.93 (0.52–1.66)		24 (43.6)	0.3	34 (45.9)	0.5	0.72 (0.34–1.55)		0.93 (0.48–1.76)	
Relationship type														
Regular	76	1.1	79	1.1	1.16 (0.68–1.98)		27 (35.5)	0.4	37 (46.8)	0.5	0.80 (0.41–1.58)		0.19 (0.11–0.34)	***
Casual – recurrent	199	2.8	117	1.7	1.75 (1.19–2.60)	**	54 (27.1)	0.8	55 (47.0)	0.8	0.90 (0.52–1.56)		0.44 (0.28–0.70)	**
Casual – new	367	5.2	144	2.1	2.21 (1.55–3.14)	***	102 (27.8)	1.5	55 (38.2)	0.8	1.62 (1.00–2.62)	*	0.53 (0.37–0.77)	**
Intercourse type														
IAI	467	6.6	173	2.5	2.74 (1.95–3.84)	***	146 (31.3)	2.1	75 (43.4)	1.1	1.67 (1.00–2.78)	*	0.54 (0.41–0.71)	***
RAI	243	3.5	•	•			42 (17.3)	0.6	•	•				
Vaginal	•	•	303	4.3			•	•	117 (38.6)	1.7				

*N*, number of intercourse events; Rate, average count per 4 weeks person-observation.

* *P* < 0.05.

** *P* < 0.01.

*** *P* < 0.001.

† Crude incidence rate ratio of male and female event counts using generalized estimating equation on paired individual data with a negative binomial link function and exchangeable correlation matrix.

‡ Crude odds ratio using generalized estimation equations on paired individual data with a logit link function and exchangeable correlation matrix.
